# Prediction of osteoporosis in patients with rheumatoid arthritis using machine learning

**DOI:** 10.1038/s41598-023-48842-7

**Published:** 2023-12-09

**Authors:** Chaewon Lee, Gihun Joo, Seunghun Shin, Hyeonseung Im, Ki Won Moon

**Affiliations:** 1 Department of Convergence Security, Kangwon National University, Chuncheon, South Korea; 2 Interdisciplinary Graduate Program in Medical Bigdata Convergence, Kangwon National University, Chuncheon, South Korea; 3 Department of Computer Science and Engineering, Kangwon National University, Chuncheon, South Korea; 4 Division of Rheumatology, Department of Internal Medicine, Kangwon National University Hospital, Chunchoen, South Korea; 5 Department of Internal Medicine, Kangwon National University School of Medicine, 1 Kangwondaehak-gil, Chuncheon, 24341 South Korea

**Keywords:** Computational biology and bioinformatics, Rheumatology

## Abstract

Osteoporosis is a serious health concern in patients with rheumatoid arthritis (RA). Machine learning (ML) models have been increasingly incorporated into various clinical practices, including disease classification, risk prediction, and treatment response. However, only a few studies have focused on predicting osteoporosis using ML in patients with RA. We aimed to develop an ML model to predict osteoporosis using a representative Korean RA cohort database. The KORean Observational study Network for Arthritis (KORONA) database, established by the Clinical Research Center for RA in Korea, was used in this study. Among the 5077 patients registered in KORONA, 2374 patients were included in this study. Four representative ML algorithms were used for the prediction: logistic regression (LR), random forest, XGBoost (XGB), and LightGBM. The accuracy, F1 score, and area under the curve (AUC) of each model were measured. The LR model achieved the highest AUC value at 0.750, while the XGB model achieved the highest accuracy at 0.682. Body mass index, age, menopause, waist and hip circumferences, RA surgery, and monthly income were risk factors of osteoporosis. In conclusion, ML algorithms are a useful option for screening for osteoporosis in patients with RA.

## Introduction

Osteoporosis is a serious health problem in patients with rheumatoid arthritis (RA). The prevalence of osteoporosis in patients with RA is approximately two times higher than that in the general population^1^. A cohort study reported that osteoporosis was present in approximately 30% of patients with RA, whereas the proportion of age- and sex-matched controls with osteoporosis was 17%^2^. Bone fragility in RA results from complex mechanisms, such as inflammatory cytokine production, osteoclast activation, and use of glucocorticoids for RA treatment. Therefore, it is crucial to detect osteoporosis in patients with RA to prevent osteoporotic fractures. Bone mineral density (BMD) measurement using dual-energy X-ray absorptiometry (DXA) is a standard diagnostic tool for osteoporosis. However, a significant number of patients with RA do not undergo a BMD measurement test in clinical practice. According to the Consortium of Rheumatology Researchers of North America registry, only 11% of patients underwent DXA during the first year of follow-up^3^. It would be thus useful to predict osteoporosis in patients with RA who are at high risk. Recently, machine learning (ML) algorithms have been extensively applied to various clinical practices, including disease classification, risk prediction, and treatment response. Although studies on the prediction of osteoporosis or fractures using ML models in the general population have been conducted, those on patients with RA are lacking. Therefore, we aimed to develop an ML model to predict osteoporosis using a representative Korean RA cohort database. This study aimed to predict osteoporosis in patients with RA using clinical data. The contributions of our paper are summarized as follows:

- Our study is the first to investigate osteoporosis prediction models in patients with RA.
- We apply the four representative ML algorithms and show that their prediction performance is comparable to previous studies on the general population, confirming the effectiveness of ML for osteoporosis prediction in patients with RA.
- By considering 83 features and their importance, we found new predictive factors like socioeconomic status, including monthly income and education, which have not been considered well before.

## Methods

### Data source and participants

**Figure 1:**
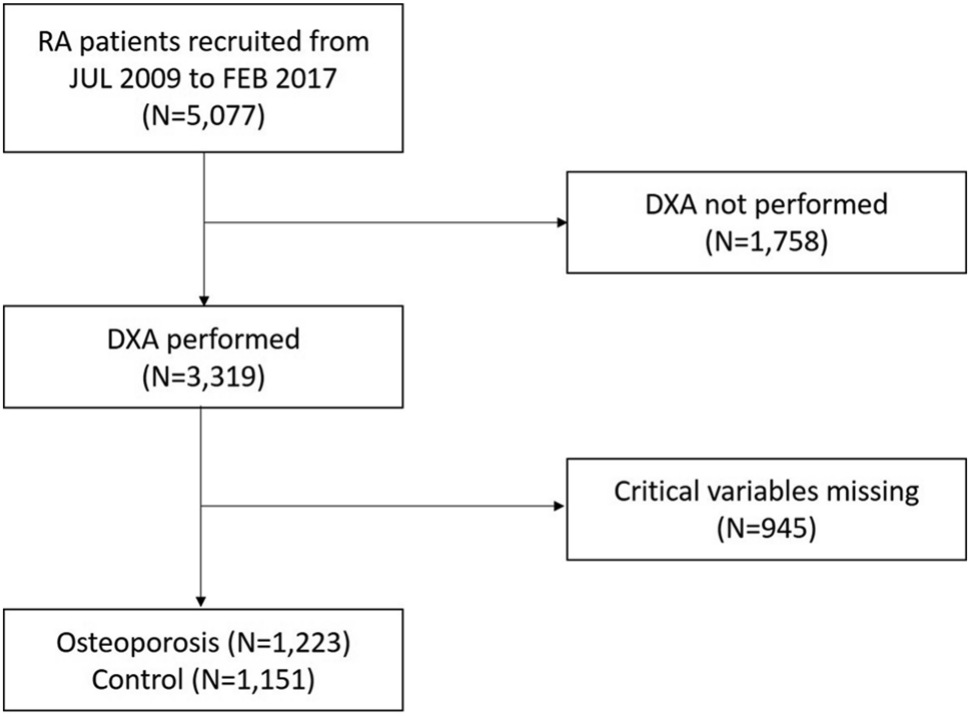
Flow diagram of study population.

The data used in this study were obtained from the KORean Observational study Network for Arthritis (KORONA) database established by the Clinical Research Center for RA in Korea^4^. KORONA includes a cohort of patients with RA recruited between July 2009 and Mar 2012. Individuals with RA who fulfilled the 1987 American College of Rheumatology classification criteria for RA and who were older than 18 years were enrolled at 23 centers in South Korea. A total of 5077 patients (4327 women, 750 men) were registered at baseline and underwent annual follow-up evaluations. All participants were provided informed consent prior to enrollment in the study. Among them, 1758 patients who had never undergone DXA were excluded. The dataset consisted of clinical information at initial enrollment as well as annual follow-up data for 5 years. Missing values of continuous features for each patient were replaced with their mean values across all follow-up data, and missing values of categorical features for each patient were replaced with their most frequent values across all follow-up data. When there was no most frequent value, that patient’s data was excluded. After this preprocessing, the total number of patients considered in the study was 2374 (Fig. 1), of which 2118 patient records were imputed.

### BMD measurement

BMD measurements were conducted using the Hologic QDR (Waltham, MA, USA) and GE Lunar Prodigy (Madison, WI, USA) systems in accordance with the standard scan and positioning protocols of the manufacturers. Osteoporosis was defined as an L-spine total or hip total value of − 2.5 or less, in accordance with the World Health Organization (WHO) classifications^5^. We classified patients with RA into osteoporosis and control groups based on their BMD values.

### Feature selection

In the KORONA database, there are over 1000 features. Principal component analysis (PCA) was performed to prevent multicollinearity. PCA was performed by grouping continuous features with correlations of  ≥ 0.7. The distribution of some representative variables obtained by PCA is shown in Supplementary Fig. 1. Continuous variables were normalized using min–max normalization. Finally, 83 features were selected for the development of the prediction models. A list of selected features is presented in Supplementary Table S1.

### Model development

Prediction was performed using four representative ML algorithms: logistic regression (LR), random forest (RF)^6^, XGBoost (XGB)^7^, and LightGBM (LGBM)^8^. LR is a model that uses regression to predict the probability of data falling into a category and classifies it as belonging to a more likely category. We used LR as the baseline method. RF is a bagging-based ensemble model composed of multiple decision trees and uses the most common value among the predicted values made by those decision trees as the final prediction. RF is robust and interpretable, and generally avoids overfitting, but is computationally expensive. XGB is an ensemble algorithm based on gradient boosting that uses a combination of multiple decision trees. It is faster than existing boosting models because it enables parallel learning on a general boosting model that combines multiple models. It is also generally more efficient than RF, though it tends to be more prone to overfitting. LGBM uses a leaf-centered tree-splitting method instead of the general balanced tree-splitting method used in gradient boosting machine models. It is typically faster and more memory-efficient than XGB. However, it is more susceptible to overfitting and can be harder to interpret.

For hyperparameter tuning, we employed GridSearchCV for LR and RandomizedSearchCV for RF, XGB, and LGBM, using fivefold cross-validation^9^. Specifically, the dataset was randomly partitioned into five subsets for each hyperparameter combination and model. In each iteration, one subset was used for testing while the remaining four as the training set. Each model underwent five evaluations, each time utilizing a different subset as the test set. The average result from the five iterations was then used as the final performance for that particular hyperparameter combination. Finally, we selected the optimal hyperparameter combination for each model and re-evaluated their performance using another fivefold cross-validation for comparison.

We tuned the following hyperparameters. For the LR model, the regularization intensity (C) was adjusted. For the RF model, max_depth (maximum tree depth), n_estimators (number of decision trees), min_samples_split (minimum number of samples required to split a node), and min_samples_leaf (minimum number of samples required to form a leaf note) were adjusted. For the XGB model, eta (learning rate), gamma (minimum loss reduction necessary to create additional partitions at the leaf nodes of the tree), max_depth, subsample (subsample ratio of training instances), and colsample_bytree (subsample ratio of columns for building each tree) were adjusted. For the LGBM model, learning rate, max_depth, subsample, and colsample_bytree were adjusted.

The accuracy, F1 score, and area under the receiver operating characteristic (ROC) curve (AUC) of each model were measured using fivefold cross-validation as mentioned above. The AUC was primarily used to compensate for the limitations of the accuracy when class distributions were different. The F1 score is the harmonic mean of precision and recall and is often used when the class distribution is imbalanced.

### Statistical analysis

Continuous and categorical variables are presented as mean ± standard deviation and number (%), respectively. Baseline characteristics between the two groups were compared using Student’s t-test for continuous variables and the chi-square test for categorical variables. Statistical significance was defined as a p-value of < 0.05. The default value of 0.5 was used for the threshold of the F1 score. Statistical analyses were performed using Python 3.8.8.

### Ethical consideration

This study was conducted in compliance with the World Medical Association Declaration of Helsinki and approved by the Ethical Review Board of the Kangwon National University Hospital (IRB approval No. KNUH-2022-03-021).

## Results

### Demographic and clinical features

Baseline clinical characteristics of both groups are shown in Table 1. Compared with the control group, the proportion of women and age were higher, BMI was lower, and previous fracture history was higher in the osteoporosis group. The proportion of current smokers and drinkers was higher in the control group. There was no statistically significant difference in oral glucocorticoid use between the two groups.

**Table 1:** Baseline characteristics for the control and osteoporosis groups.

Variables	Control (N = 1151)	Osteoporosis (N = 1223)	*P* value
Female sex	1024 (89)	1146 (94)	< 0.05
Age (years)	54.81 ± 1.05	62.45 ± 0.9	< 0.05
Body mass index (kg/m^2^)	23.09 ± 0.29	22.09 ± 0.2	< 0.05
Previous fracture	117 (10)	243 (20)	< 0.05
Family history of RA	98 (9)	108 (9)	0.84
Current smoking	85 (7)	51 (4)	< 0.05
Current drinking	221 (19)	193 (12)	< 0.05
Oral glucocorticoid use	837 (73)	921 (75)	0.16

Values denote number (%) or mean** ± **standard deviation unless stated otherwise.

### Model evaluation

**Figure 2:**
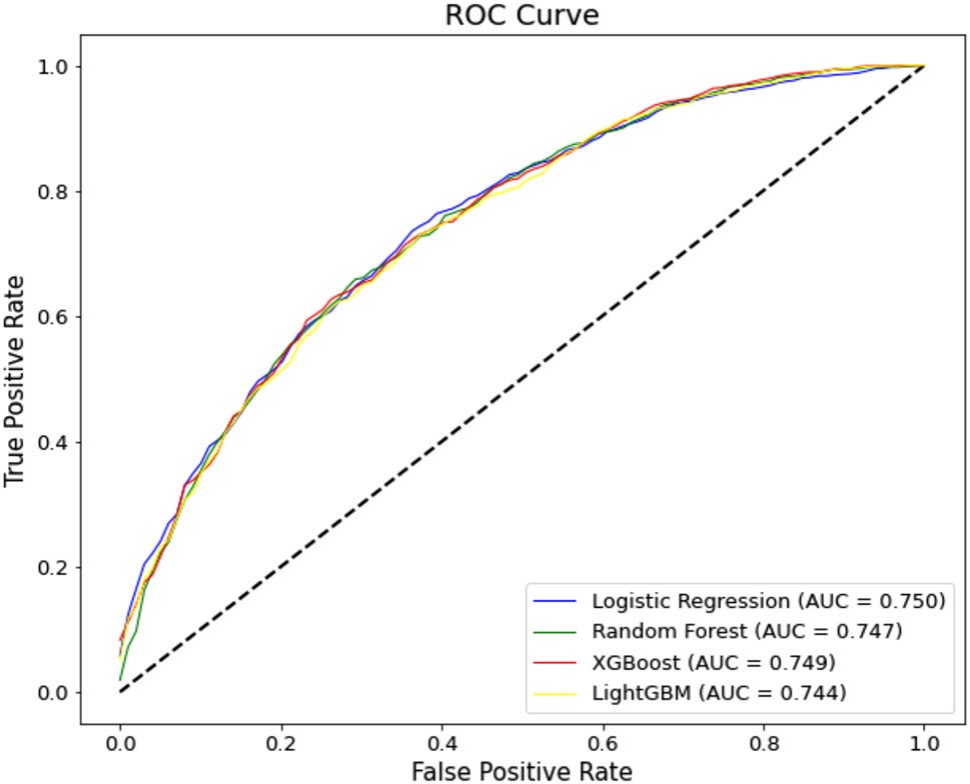
Receiver operating characteristic (ROC) curves for the prediction models.

The AUC, accuracy, and F1 score of each model were measured using fivefold cross-validation after selecting an optimal hyperparameter combination for each model using either GridSearchCV or RandomizedSearchCV. The performance of each model in predicting osteoporosis using 83 clinical features is shown in Table 2, with 95% confidence intervals for the AUC. The AUC was highest in the LR model at 0.750, the accuracy was highest in the XGB model at 0.682, and the F1 score was highest in the RF model at 0.70, although the difference between the models was not significant. Figure 2 shows the ROC curve for each ML model.

**Table 2:** Performance of each prediction model using fivefold cross-validation, with 95% confidence intervals for the AUC.

Model	AUC	Accuracy	F1 score
Logistic regression	0.750 (0.717–0.783)	0.681	0.700
Random forest	0.747 (0.715–0.781)	0.681	0.705
XGBoost	0.749 (0.72–0.779)	0.682	0.700
LightGBM	0.744 (0.715–0.772)	0.678	0.693

### Intraclass correlation coefficient (ICC)

**Figure 3:**
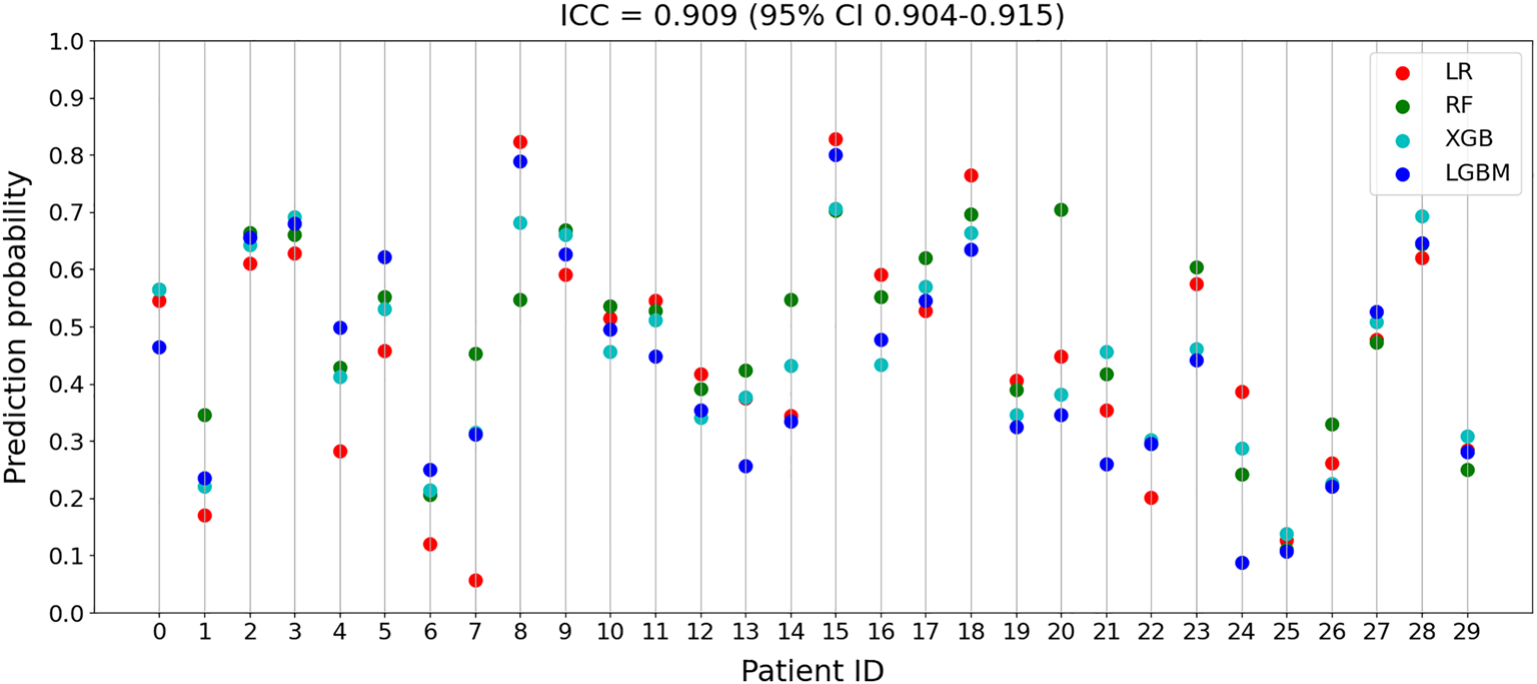
Intraclass correlation of part of the dataset using ICC (3, 1).

We also calculated the ICC^10^ to see the prediction consistency between the ML models for each patient. We considered the ML models as a fixed set of raters, their prediction probabilities as ratings, and the patients as targets. The ICC value ranges from 0 to 1, with values between 0.75 and 0.9 indicating good reliability and values greater than 0.90 indicating excellent reliability^10^. Figure 3 shows the prediction probabilities of each ML model for a sample of 30 out of 2374 patients, together with the ICC (3,1) value of 0.909 (95% CI 0.904–0.915), which indicates that the ML models are highly consistent and reliable.

### Feature importance of each model

**Figure 4:**
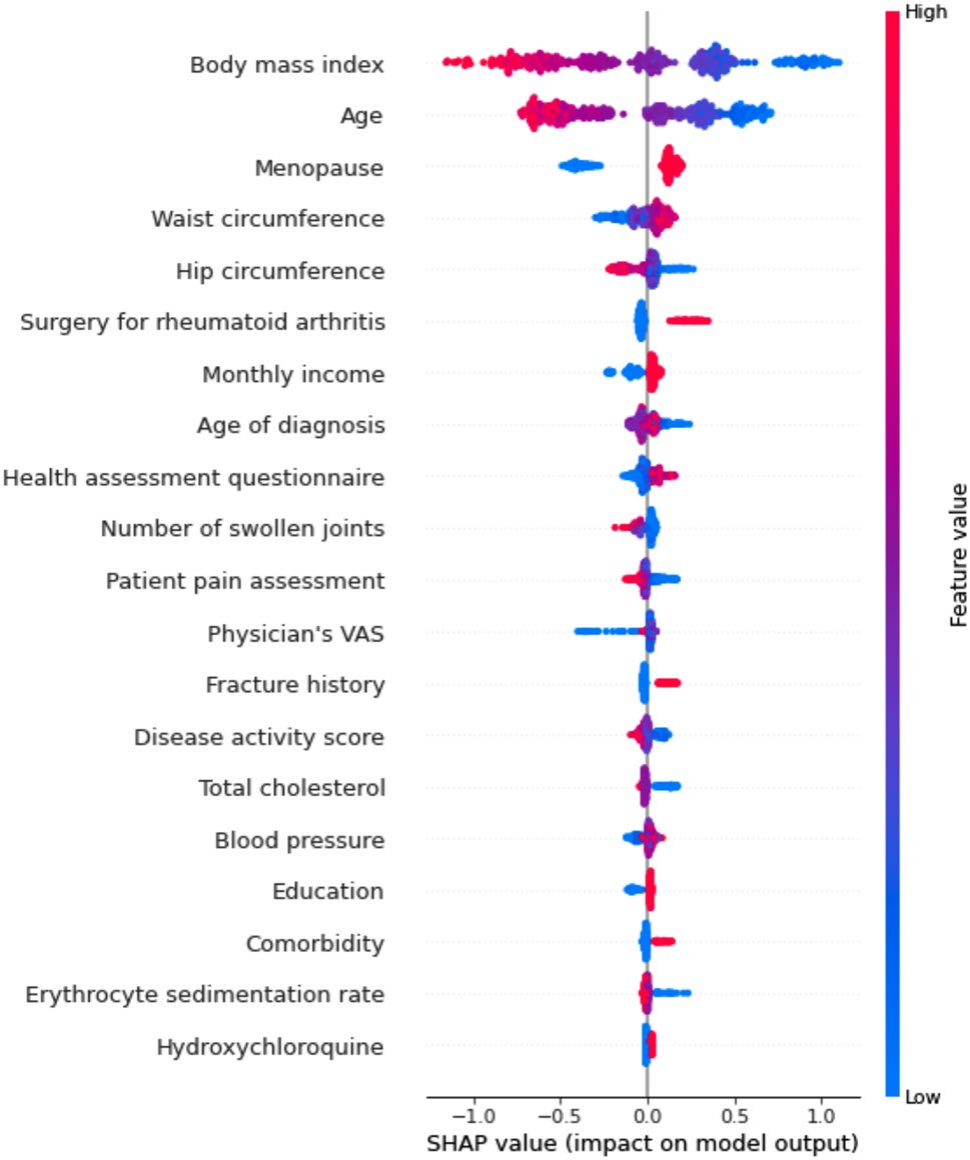
Top 20 features derived from the XGBoost model.

The feature importance of each model was calculated based on the fold with the highest AUC among the five folds used for cross-validation. The LR model was not considered because it was solely used as a benchmark to evaluate the efficacy of tree-based prediction models and did not fully resolve the issue of multicollinearity among the input features. The 20 most important features of the XGB model are shown in Fig. 4. The top 20 most important features of the other models are shown in Supplementary Figs. 2 and 3.

## Discussion

We developed and evaluated ML models to predict osteoporosis risk in patients with RA. Osteoporotic fracture is a serious health issue in patients with RA. A British study showed that the relative risk (RR) of hip fracture of patients with RA was 2.0 (95% CI 1.8–2.3) and that of vertebral fracture was 2.4 (95% CI 2.0–2.8)^11^. These fragility fractures impair quality of life and increase healthcare costs and mortality rates. Korean national health claims data reported that the incidence of osteoporotic fractures is higher in patients with RA than in the general population over 3 years (17.4% vs. 11.6%), and the standardized mortality rate was 1.4 times higher in men and 1.3 times in women^12^. However, timely screening and appropriate management of osteoporosis remain inadequate in clinical practice. A retrospective study from the USA reported that only 30% of female patients with RA underwent DXA during 4.4 years^13^. A French study showed that DXA was performed in 21.6% of 1008 patients with RA, and approximately 12% of patients received osteoporosis medication^14^. A large observational study from the USA of 11,669 patients with RA followed from 2003 to 2014 revealed that approximately 50% of patients who required treatment did not receive medication for osteoporosis^15^. Identification of osteoporosis in high-risk patients with RA is an important issue in clinical practice.

Many researchers have focused on developing osteoporosis prediction tools using ML for the general population. Table 3 shows the characteristics and results of studies on osteoporosis prediction in the general population. The AUC scores in these studies ranged between 0.710 and 0.854. The AUC score in our study was 0.750, which is a relatively good performance compared with other studies. Other studies reported that the best performing models were artificial neural networks, XGB, RF, and multilayer perception models. Our results showed that the LR model had the best performance, whereas the other models showed similar performances. To the best of our knowledge, no studies have been conducted on the prediction of osteoporosis in patients with RA until now. Recently, one study investigated the prediction of fractures with ML in elderly patients with RA. Chen et al.^16^ reported an ML model to predict the risk of fractures in patients with elderly-onset RA. They collected data from 487 elderly patients with RA and developed various ML models for fracture risk. The AUC of each model was 0.713–0.872.

**Table 3:** Key studies on the osteoporosis prediction using machine learning.

Study	Number of patients	Number of selected features	Best performance model	Best performance (AUC)
Shim et al.^20^	1792	9	ANN	0.743
Erjiang et al.^21^	13,577	30	XGB	0.833
Yang et al.^22^	5982	16/19	RF	0.843/0.811
Wang et al.^23^	1419	18	ANN	0.762
Iliou et al.^24^	3426	2	MLP	0.710
Park et al.^19^	3309	20	XGB	0.730/0.790
Bui et al.^25^	1951	15	RF	0.854
Our study	2374	83	RL	0.750

*ANN* Artificial neural network; *LR* Logistic regression, *MLP* Multi-layer perceptron, *RF* Random forest, *XGB* XGBoost.

In RA, conventional risk factors for osteoporosis include female sex, smoking, old age, low BMI, menopause, diabetes, thyroid disorders, lack of physical activity, and glucocorticoid use^17^. Lee et al.^18^ investigated risk factors for osteoporosis using a traditional statistical technique and the same database used in our study, the KORONA registry. In Lee et al.’s study, the number of patients with osteoporosis (n = 619) was smaller than that in our study (n = 1223) as enrolled patients were limited to postmenopausal women and men over 50 years old. The clinical characteristics were quite different between Lee et al.’s study and the present study. For example, in our study, the proportion of current smokers and drinkers was higher in the control group as this group included more male patients. We intended to develop a prediction model regardless of sex; therefore, we included both male and female patients. Lee et al. reported that older age, lower BMI, longer disease duration, higher cumulative glucocorticoid dose, and higher health assessment questionnaire scores were independent risk factors of osteoporosis^18^. However, our ML model suggested new features such as monthly income, education, surgical history, and marital status as predictive factors of osteoporosis. Interestingly, socioeconomic status, including monthly income and education, was selected as a predictive factor in addition to previously well-known conventional predictors. Another Korean study that developed an ML model using National Health and Nutrition Examination data showed that monthly income was an important predictive factor of osteoporosis^19^. Table 4 shows a comparison of the top 10 ranked features from other studies and our study. In most studies, age and BMI were identified as the highest-ranked predictive features. Menopause, bisphosphonates, and estrogen use were also ranked high. In addition to conventional risk factors, features including alkaline phosphatase, cholesterol, uric acid, and blood pressure are important for prediction. These findings provide new insights into the prediction of osteoporosis in clinical practice.

**Table 4:** Comparisons with the top 10 ranked features from other studies on osteoporosis prediction using machine learning.

Ranking	Erjiang et al.^21^	Park et al.^19^	Bui et al.^25^	Our study
1	Age	Age	Age	Body mass index
2	Weight	Menopause	Weight	Age
3	Bisphosphonate use	Alkaline phosphatase	Height	Menopause
4	Body mass index	Weight	Uric acid	Waist circumference
5	Denosumab use	Forced vital capacity	Calcium	Hip circumference
6	Estrogen use	Sobriety	Cholesterol	Surgery for RA
7	Chronic respiratory disease	Aspartate aminotransferase	Creatinine	Monthly income
8	Osteopenia	Diastolic blood pressure	Free thyroxine level	Age of diagnosis
9	Height loss	Riboflavin intake	Glucose	Health assessment questionnaire
10	Hormonal therapy	Weight control by exercise	HbA1c	Number of swollen joints

Our study had several strengths. First, we developed a prediction model for patients with RA who have not `been covered in previous research. The performance of our model was comparable to that of previous studies on the general population. Second, our study used 83 clinical features for the development of the prediction models, which is much greater than the number of features used in other studies. We selected these features from more than 1000 features using PCA to avoid multicollinearity problems. However, our study has the following limitations. First, there was no separate dataset to validate this model. We could not find any RA patient cohort dataset that included BMD results. However, the KORONA data were collected from 23 separate hospitals in Korea, and heterogeneity existed in the dataset. Second, we could not develop a fracture-prediction model. Although clinical information on fractures was available, the number of fracture events was too small to create a prediction model.

In conclusion, we applied representative ML algorithms to predict osteoporosis using clinical data from patients with RA. By comparing with previous studies, we observed a comparable performance. ML methods have the potential to support practitioners in the detection of osteoporosis in patients with RA.

### Supplementary Information

Supplementary Information.

## Data Availability

Raw data were generated by the Clinical Research Center for RA in Korea. The data supporting the findings of this study are available from the corresponding authors upon request.

## Code availability

The code for experiments is available at https://github.com/knu-plml/RA-osteoporosis-ml-prediction.
