# Supplementary material for: Prediction of osteoporosis in patients with rheumatoid arthritis using machine learning
Source: Sci Rep. 2023 Dec 9;13:21800. doi: 10.1038/s41598-023-48842-7 (PMC10709305; doi:10.1038/s41598-023-48842-7)

**Supplementary Table 1. Features used for the development of the ML prediction models.**

|  | Feature |
| --- | --- |
| Clinical features | Gender |
|  | Age |
|  | Age of diagnosis |
|  | Number of American College of Rheumatology classification criteria satisfied at first diagnosis |
|  | Physician’s visual analogue scale |
|  | Height |
|  | Weight |
|  | Body mass index |
|  | Waist Circumference |
|  | Hip Circumference |
|  | Blood pressure |
|  | Presence of tender joint |
|  | Presence of swollen joint |
|  | Number of tender joints |
|  | Number of swollen joints |
|  | Family history of rheumatoid arthritis |
|  | Compliance with dose |
|  | Adverse drug effect |
|  | Combined diseases (Cardiovascular system, respiratory system, digestive system, renal urinary system, other diseases) |
|  | Rheumatoid arthritis with surgical experience |
|  | Fracture history |
|  | Monthly income |
|  | Marital status |
|  | Education |
|  | Regular exercise |
|  | Current smoking status |
|  | Current alcohol drinking |
|  | Morning stiffness |
|  | Patient pain assessment |
|  | Use of alternative medicines |
|  | Menopause |
|  | Disease activity score |
|  | Health assessment questionnaire |
|  | Euroqol-5 dimension |
| Medication | Methotrexate |
|  | Hydroxychloroquine |
|  | Sulfasalazine |
|  | Leflunomide |
|  | Bucillamine |
|  | Mizoribine |
|  | Tacrolimus |
|  | Azathioprine |
|  | Cyclosporine |
|  | Oral glucocorticoid |
| Laboratory results | White blood cell |
|  | Hemoglobin |
|  | Hematocrit |
|  | Platelet |
|  | Alanine aminotransferase |
|  | Aspartate aminotransferase |
|  | Alkaline Phosphatase |
|  | Blood urea nitrogen |
|  | Creatinine |
|  | Total cholesterol |
|  | Erythrocyte sedimentation rate |
|  | C-Reactive protein |

**Supplementary Figure 1. Distribution of some representative dimensionally reduced variables of the control and osteoporosis groups after principal component analysis.**


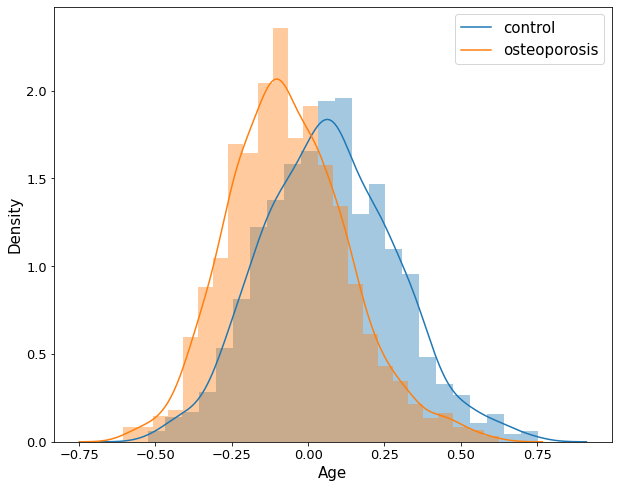

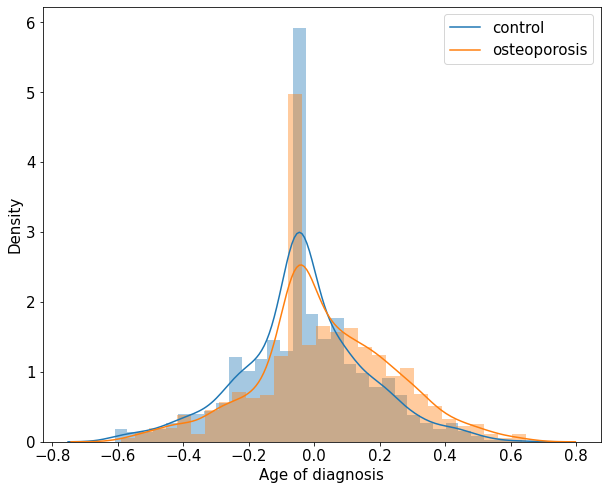

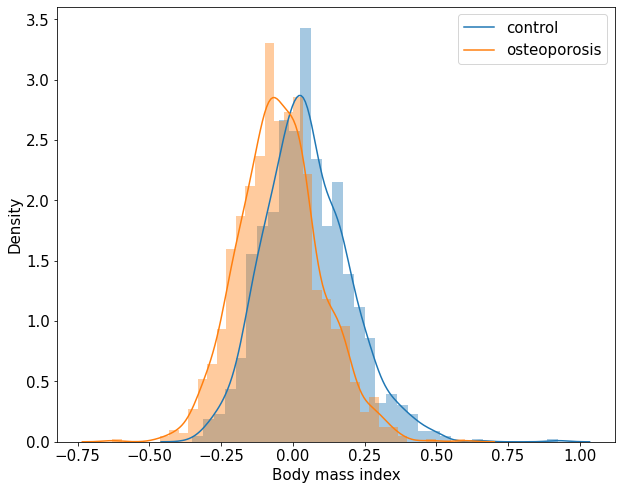

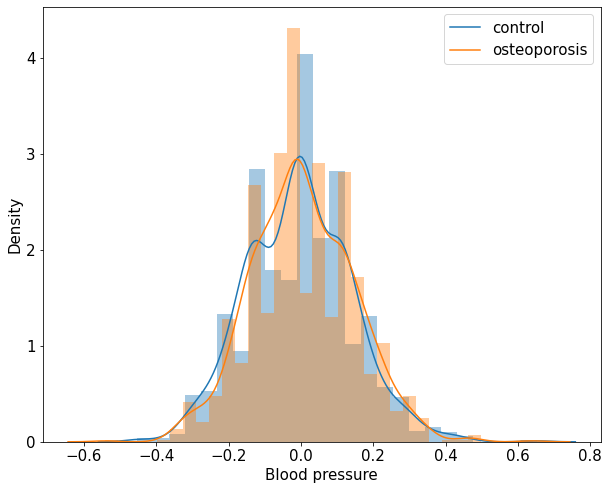

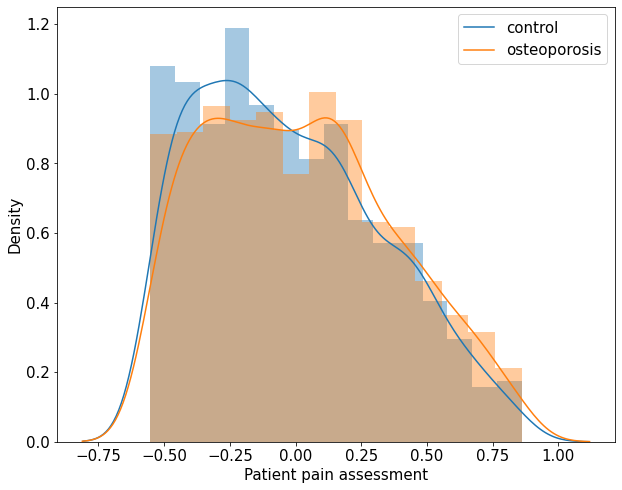


**Supplementary Figure 2. Top 20 features derived from the Random Forest model.**


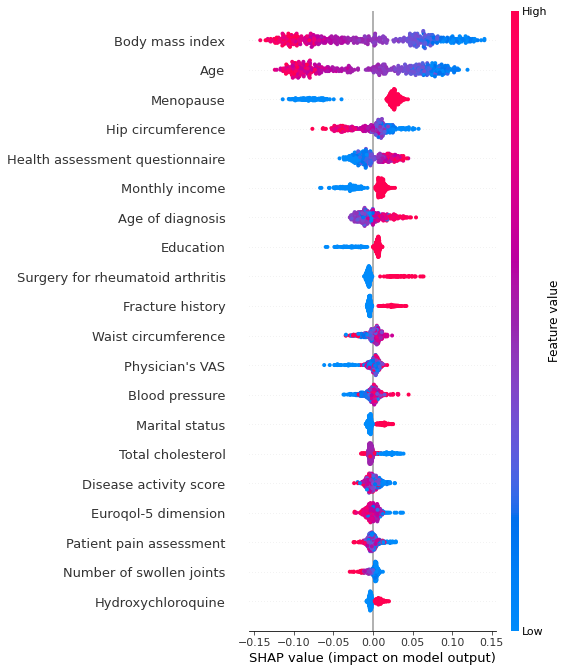


**Supplementary Figure 3. Top 20 features derived from the LightGBM model.**


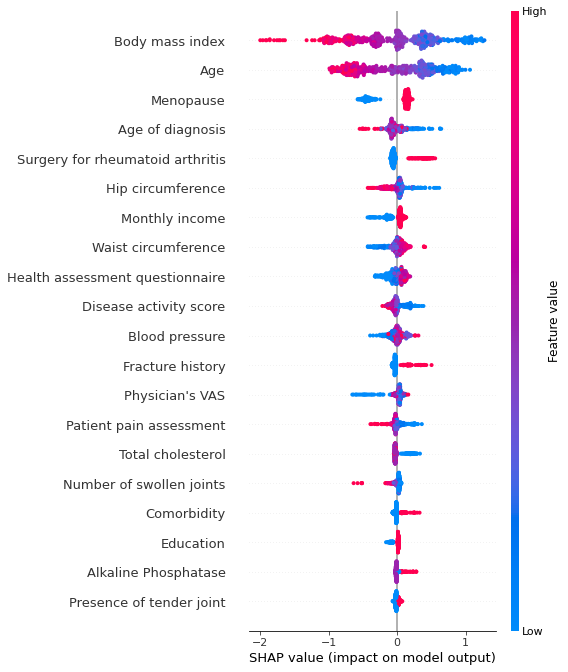

Supplement: Supplementary file 1 — Supplementary Information. [file 41598_2023_48842_MOESM1_ESM.docx]
